# SkipCor: Skip-Mention Coreference Resolution Using Linear-Chain Conditional Random Fields

**DOI:** 10.1371/journal.pone.0100101

**Published:** 2014-06-23

**Authors:** Slavko Žitnik, Lovro Šubelj, Marko Bajec

**Affiliations:** 1 Laboratory for Data Technologies, University of Ljubljana and Optilab d.o.o., Ljubljana, Slovenia; 2 Laboratory for Data Technologies, University of Ljubljana, Ljubljana, Slovenia; University of Illinois-Chicago, United States of America

## Abstract

Coreference resolution tries to identify all expressions (called mentions) in observed text that refer to the same entity. Beside entity extraction and relation extraction, it represents one of the three complementary tasks in Information Extraction. In this paper we describe a novel coreference resolution system SkipCor that reformulates the problem as a sequence labeling task. None of the existing supervised, unsupervised, pairwise or sequence-based models are similar to our approach, which only uses linear-chain conditional random fields and supports high scalability with fast model training and inference, and a straightforward parallelization. We evaluate the proposed system against the ACE 2004, CoNLL 2012 and SemEval 2010 benchmark datasets. SkipCor clearly outperforms two baseline systems that detect coreferentiality using the same features as SkipCor. The obtained results are at least comparable to the current state-of-the-art in coreference resolution.

## Introduction

The field of Information Extraction (IE) deals with automatic extraction of structured information such as person names, locations, organizations etc. from unstructured or semi-structured text. The roots of IE dates back to 1970s when the first approaches emerged [1]. Since then a lot of effort has been put in finding solutions that would facilitate efficient and accurate IE. This has resulted in many different IE systems that are available today or at least described in the literature [2], [3]. Nevertheless, we are still not able to extract information with high precision and recall especially when performing IE on large unstructured datasets such as on the web for example. This and the fact that the amount of unstructured data is rapidly growing make the IE field more and more important.

The IE task can be divided into three subtasks: entity extraction, relation extraction and coreference resolution. As its name implies, the entity extraction focuses on the extraction of entities, i.e. parts of the text or expressions in text that can be categorized as one of the predefined categories, such as the names of places, persons, organizations, dates, etc. The relation extraction then seeks to identify relations among the identified entities (e.g. some expression that was identified as a name of a person is found to be related to some other expression identified as an organization). Finally, the coreference resolution tries to identify parts of the text or expressions that refer to the same entity in the analyzed text. The expressions that we observe in the coreference resolution are called “mentions” and can be one of the following types: named mentions (e.g. “*John Doe* was here”), nominal mentions (e.g. “*the boy* was here”) or pronominal mentions (e.g. “*he* was here”) [4]. To detect mentions that refer to the same entity, a two-steps procedure is usually performed: (1) the identification of all mentions in the observed text and (2) the clustering of the identified mentions so that all mentions referring to the same entity fall into the same cluster.

Coreference resolution [5] represents an important step in IE as it provides bases for merging contextual information extracted through other tasks [6]. For example relations and attributes that are identified in entity [7] and relation extraction and are associated with a particular mention hold not only for that particular mention but also for other coreferent mentions, even the distance between them is several words or sentences [8]. The identification of coreferent mentions in text has already proved useful in various domains, ranging from mining news articles [9] to biological data [10].

In this paper we describe a novel coreference resolution system ‘SkipCor’, which is based on the well known conditional random fields algorithm [11]. The novelty of SkipCor lies in a special transformation of input data into the so called *n* skip-mention sequences, in which only every (*n+1*)-th mention is included. This allows the use of very simple first-order (i.e., linear-chain) models that enable much faster and exact training and inference than do the general models. Thus, in contrast to most other approaches, the proposed system is completely parallelizable with a linear time complexity (in the number of mentions in the text). We compare SkipCor to a baseline system, on seven standard benchmark datasets. It clearly outperforms the baseline system that uses only a single sequence of mentions and a standard pairwise system that, as in traditional approaches mentioned above, looks at all the mention pairs in order to identify the coreferent ones. Furthermore, the results obtained are at least comparable to the current state-of-the-art in coreference resolution. We also investigate the drop in accuracy to be expected in real-world scenarios, where systems are trained on one dataset, and adopted on another, something which may be of independent interest.

## Background

The majority of techniques for coreference resolution transform the problem into a pairwise classification task [12], [13] (i.e., the algorithm checks every pair of mentions for coreference). This enables the use of standard machine learning classifiers that rely on hand-labeled data sets. On the other hand, unsupervised techniques infer the coreferentiality based on sequences of mentions [14], [15], which are much harder to train and are not easily generalized to new problems or domains. In this section we will provide an overview of the different coreference resolution systems, with special focus on approaches based on graphical models [11] (as SkipCor).

One of the earliest supervised approaches used a decision tree algorithm and twelve informative feature functions [16]. That approach was the first to improve on the performance of previously state-of-the-art rule-based techniques. Even though the adopted features were based solely on pairs of mentions with local information, it was difficult to improve their results by only using more sophisticated algorithms. Therefore, a number of innovative and linguistic-rich feature functions [13], [17] along with different algorithms like maximum entropy [18], SVM classifiers [19] and Markov Logic Networks [20] have been proposed in the recent literature. Recently, Bengston and Roth [17] have systematically divided different feature functions into categories and clearly demonstrated their importance. In particular, they have shown that the development of well-designed features can greatly improve the performance of a coreference resolution system. Due to the similarities among the proposed supervised systems, the Reconcile platform [21] was developed in order to provide a common framework for new algorithms, features, and their evaluation.

Unsupervised approaches demand no training data. Nevertheless, unsupervised state-of-the-art systems still achieve comparable results to the supervised systems. Haghighi and Klein [15] proposed a modular unsupervised system using rich features. The system is based on a three-step procedure, consisting of the extraction of syntactic paths from the mentions, the evaluation of semantic compatibility between the mentions, and the selection of reference mentions, which serve as the basis for using pairwise decisions over transitive closures. Lee et al. [14] upgraded Raghunathan's system [22], which is based on a multi-pass sieve approach. They employed thirteen sieves (i.e., sequential processing steps) sorted by precision. During the execution of each sieve, the entire dataset is processed by applying a few manually written patterns. These hand-crafted patterns relate only to syntactic parse trees and extracted named entities, and are based on different heuristics and dataset specifications. Some unsupervised techniques have also been proposed. They infer coreferentiality based on sequences of mentions [23]–[25].

In the field of factor graphs, McCallum et al. [26] proposed three general conditional random fields (CRF) models to solve the coreference resolution problem. The first is a general model (i.e., the CRF structure is unrestricted) and the training or inference is therefore complex. In such cases exact inference is not possible and therefore approximation algorithms must be used to compute right marginal values for the underlying CRF structure [27]. The second model represents pairs of mentions by specific attributes, while the third represents the pairs as nodes in the model. Wellner et al. [28] successfully applied coreference resolution to citation matching, interestingly by using a special case of McCallum's first model combined with named entity extraction. Most similar to the linear models, a skip-chain CRF has been proposed in [29], which also supports the use of long-distance dependencies by incorporating additional cliques into the model. Still, longer times are needed for training and inference compared to linear-chain CRF. Cullota et al. [12] proposed the use of first-order probabilistic models over sets of mentions; thus, the algorithm operates directly on the entities. To avoid a combinatorial explosion of all possible entity subsets, they incrementally merged different mentions into sets. Later, they also included the step of canonicalization [28], which refers to the process of generating the underlying entities along with their attributes. Recently, Sundar et al. [30] proposed a CRF-based coreference resolution system. They further decomposed the problem into two subtasks: pronominal resolution using general CRFs that has only parse tree features, and non-pronominal resolution using linear-chain CRFs that has different string similarity features. Although the system is based on linear models, the input to the models still consists merely of sequences of length two.

In Table 1 we show the classification of some of the coreference resolution approaches that have been put forth in the literature. We categorize the systems along two dimensions: the type of input to the algorithm, and the type of model learning. As can be observed, the proposed SkipCor system is novel from the perspective of the selected dimensions. Among the unsupervised approaches, coreference resolution systems have been developed for both pairwise and sequence-based input types. In contrast, supervised approaches have mainly employed only pairwise comparisons. The system in [30] is similar to our baseline algorithm, SkipCorPair; however, it predicts whether two mentions are coreferent using a CRF algorithm. Also, [26] presents some CRF-based methods, but it evaluates only a version where each node represents a pair of mentions.

**Table 1:** Classification of coreference resolution approaches.

	UNSUPERVISED	SUPERVISED
**SEQUENCE-BASED**	[23]–[25]	**SkipCor**
**PAIRWISE**	[14], [15], [22], etc.	[12], [13], [16]–[20], [26], [30]

According to the two-dimensional classification of coreference resolution systems, the proposed SkipCor system solves the problem in a novel fashion.

In summary, SkipCor represents a novel CRF-based approach that identifies coreferences over mention chains and employs simple clustering to uncover all mentions in the text that refer to the same entity. In contrast to other systems, we adopt a supervised algorithm for training and inference on sequence-based data. Thus, instead of using a pairwise or set-based approach, we consider sequences of mentions in some document and use simple linear-chain CRF models. To enable the use of such simple models, we introduce an adequate transformation of the data into skip-mention sequences. Consequently, the feature functions also refer to non-local information and can detect distant mention coreferences. Note also that the training and inference of linear-chain CRFs can be solved with a fast and exact algorithm, which significantly reduces the time complexity of the system.

### Conditional Random Fields

Conditional random fields (CRF) [11] is a discriminative model that estimates the joint distribution 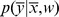 over the target sequence 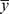 conditioned on the observed sequence 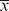 and weight vector 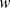 (see below). We represent a sentence by a sequence of words 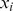 with additional corresponding sequences that represent attribute values such as part-of-speech tags 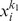, lemmas 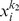, relations 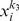, and other observable values 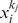. These values are used by feature functions 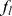 that are weighted during CRF training in order to model the target sequence 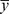. The sequence 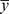 corresponds to the source sequence and consists of the labels that we would like to automatically infer. For named entity recognition, we commonly use tags such as PER for person type, ORG for organization, and LOC for location. Similarly for relationship extraction, we use tags WORKS-AT, LIVES-IN, etc. For the coreference resolution task, we build sequences containing only mentions, as opposed to sequences containing all the words in a document. Then we use the label C if the current mention is coreferent with the previous one, and O otherwise.

In the field of IE, CRFs have been successfully employed for various sequence labeling tasks and have achieved state-of-the-art results. It can also deal with a large number of multiple, overlapping, and non-independent features.

Training a CRF is thus maximizing the conditional log-likelihood of the training data, by which we find a weight vector 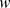 that predicts the most probable sequence 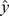 for a given 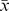. Hence,
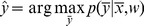
(1)where the conditional distribution is
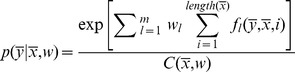
(2)


Here, 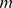 is the number of feature functions and 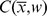 is a normalization constant computed over all possible sequences 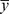.

**Figure 1:**
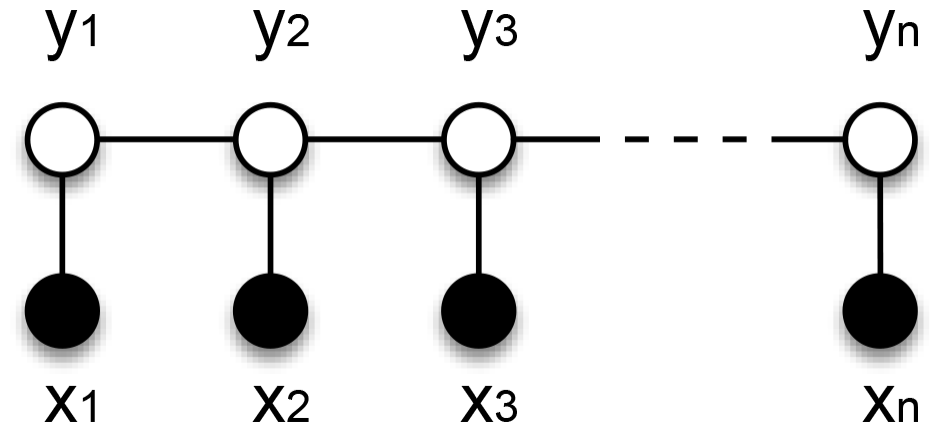
Linear-chain conditional random fields model. Black nodes represent observable values, which are in our case entity mentions. White nodes represent hidden labels that we need to predict and define whether the current observable value is coreferent with the previous one.

The structure of a CRF defines how the dependencies with target labels are modeled. A general graphical model (i.e., a graph denoting the conditional dependence structure) can depend on many labels and is therefore intractable for training or inference without complex approximation algorithms. Thus, we use only a simple linear-chain CRF (LCRF) model, which depends on the current and previous labels (i.e., a first order model). The structure of such a model is represented in Figure 1. Furthermore, with the use of a number of feature functions and special dataset transformations, our method achieves comparable results to the best known systems.

## Methods

In this section we introduce the proposed SkipCor algorithm. First, we overview and introduce new feature functions used by conditional random fields models in the present paper. Next, we explain the data representation using skip-mention sequences and illustrate the coreference resolution execution of the proposed system on an example document. We also support the proposed representation by examining the distribution of consecutive coreferent mention distances on a representative coreference dataset. Last, we explain the implementation [31] of the proposed SkipCor system and discuss the time complexity of the algorithm.

### Feature Functions

The selection of informative features is the main source of an increase of precision and recall when training machine learning classifiers. Feature functions are usually implemented as templates and the final features are then generated by scanning the entire training data. In natural language processing, a few thousand or more features are commonly used, which can be efficiently handled by a CRF. A feature function that returns 1 if the current mention is of person type or the previous mention is equal to “Mr.” and 0 otherwise, is defined by: 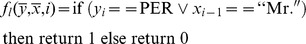



Although many feature functions have been proposed in the literature [7], [16], [17], [32]–[34], we introduce new feature functions for the purpose of this research. These can be sorted into the following categories:

#### Preprocessing

These feature functions use standard preprocessing labels, which are a result of the preprocessing step, such as lemmas, part-of-speech (POS) tags, chunks, and parse trees. The derived feature functions are “target label distribution”, “do POS tags match on distances up to two mentions away”, “distribution of POS tags”, “mention type match”, “is a mention pronoun of demonstrative/definitive noun phrase”, “is mention a pronoun”, “length between mentions within a parse tree”, “parse tree path from the root node”, “parse tree path between the two mentions”, “depth of a mention within a parse tree”, and “parse tree parent value match”.

#### Location

Sometimes it is important to know where the mention resides. Location feature functions deal with the mention's location compared to the whole document, sentence, or other mentions. Our approach already implicitly uses mention distance at each skip-mention model, but we still employ some specific feature functions. These are “sentence/mention/token distance between the two mentions”, “is first/last mention” and “are mentions within the same sentence”.

#### Mention Shape

Mention constituents are represented as word phrases and by using mention shape features we are interested in whether two of them share some property. These feature functions are string-based and are implemented as follows: “does a mention start with an upper case”, “do both mentions start with upper case”, “does a prefix/postfix/whole of left/right mention on distances up to five mentions match”, “does a mention text/extent match”, “is one mention appositive of another”, “is one mention prefix/suffix/substring of another”, “Hearst mention co-occurrence rules”, “is a mention within quotes”, “does a mention contain head/extent words of another” and “length difference between the two mentions”.

#### Semantic

This class of feature functions captures semantic relationships between mentions by employing additional semantic sources, such as WordNet [35], specialized lexicons, semantic gazeteer lists, and ontologies. The semantic feature functions are “do named entity types match”, “do mentions agree on gender/number” [36], “is one mention appositive of another”, “is a mention an alias of another” (heuristically), “edit distance similarity between two mentions”, “WordNet relation (hypernym/hyponym/synonym) between the mentions”, “do mentions share the same WordNet synset”, “current mention word sense”, “do both mentions represent an animate object” [37] and “do both mentions speak” (taking context words into account).

A brief description and exact list of feature functions that we use is presented in Table 2. Still, their exact implementations can be retrieved from our public source repository [31] (within the class FeatureFunctionPackages).

**Table 2:** Feature functions description.

Name	Description	Model
Target label distribution	Distribution of target labels.	A, S, C
Starts upper	Does the mention start with an upper case letter.	A, S, C
Starts upper twice	Do two consequent mentions start with an upper case letter.	A, S, C
Prefix value	Value of the prefix (length of 2 and 3) for the mention on offset distance (distances from −5 to 5) from the current mention.	A, S, C
Suffix value	Value of the suffix (length of 2 and 3) for the mention on offset distance (distances from −5 to 5) from the current mention.	A, S, C
Consequent value	A combination of values of the consequent mentions on offset distance (distances from −4 to 4) from the current mention.	A, S, C
String match	Do consequent mention values match.	A, S, C
Gender match	Does the gender of two consequent mentions match.	A, S, C
Gender value	The gender value of the mention.	A, S, C
Is appositive	Is the mention appositive of the another.	A, S, C
Alias	Is the mention alias or abbreviation of the another.	A, S, C
Is prefix	Is the mention prefix of the another.	A, S, C
Is suffix	Is the mention suffix of the another.	A, S, C
Similarity value	How similar are the two mention values according to the Jaro Winkler [53] metric.	A, S, C
Is pronoun	Is the mention a pronoun.	A, S, C
Same sentence	Are consequent mentions in the same sentence.	A, S, C
Hearst co-occurence [54]	Does the text between the two mentions follow some predefined rules, e.g. 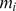 such as 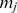 .	A, S, C
Sentence distance	What is the distance between the sentences of the two mentions.	A, S, C
Is quoted	Is the mention within the parentheses.	A, S, C
Substring match	Is the mention a substring of the another.	A
Starts with	Does the mention starts with the another.	A, S, C
Ends with	Does the mention ends with the another.	A, S, C
Number match	Do the mentions match in number (i.e., singular, plural).	A, S, C
Mention type	Type of mention (i.e., pronoun, name, nominal).	A
Relative pronoun	Heuristic decision if the mention is a relative pronoun of the another.	A
WordNet [35]	How is the mention semantically connected to the another (e.g., is a hypernym, synonym).	A
WordNet synset	Are the two consequent mentions in the same synset.	S, C
Entity type	What is the named entity type or subtype of the mention.	A
Length difference	What is the difference in length of the two consecutive mentions.	A, S, C
Is demonstrative	Is the mention a demonstrative noun phrase.	A, S, C
Offset match	Do consecutive POS values on distances from −2 to 2 match.	A
Parse tree path	Path values between the two mentions in a parse tree.	A, S, C
Parse tree mention depth	Depth of the mention within the parse tree.	A, S, C
Parse tree parent value	Parse tree value of the mention on lengths of one, two or three.	A, S, C
Relation	Does a relationship exist between the two consecutive mentions.	S
Speaker	Who is the current speaker in a transcript text.	C

The feature functions are used by all skip-mention CRF models and are modeled as unigram or bigram features. The exact details (e.g., which mention values are used by a specific feature functions) and implementations can be retrieved from our public source repository [31] (within the class FeatureFunctionPackages). The abbreviations A, S and C define which feature functions were used when training the models for the ACE2004, SemEval2010 and CoNLL2012 datasets, respectively.

### Skip-mention Sequences

Since merely linear-chain CRF models are used, we can identify only coreferences over two directly consecutive mentions. Thus, to detect coreferences over mentions on larger distances, i.e., having one, two, three, or more mentions in between, we propose a skip-mention dataset transformation.

**Figure 2:**
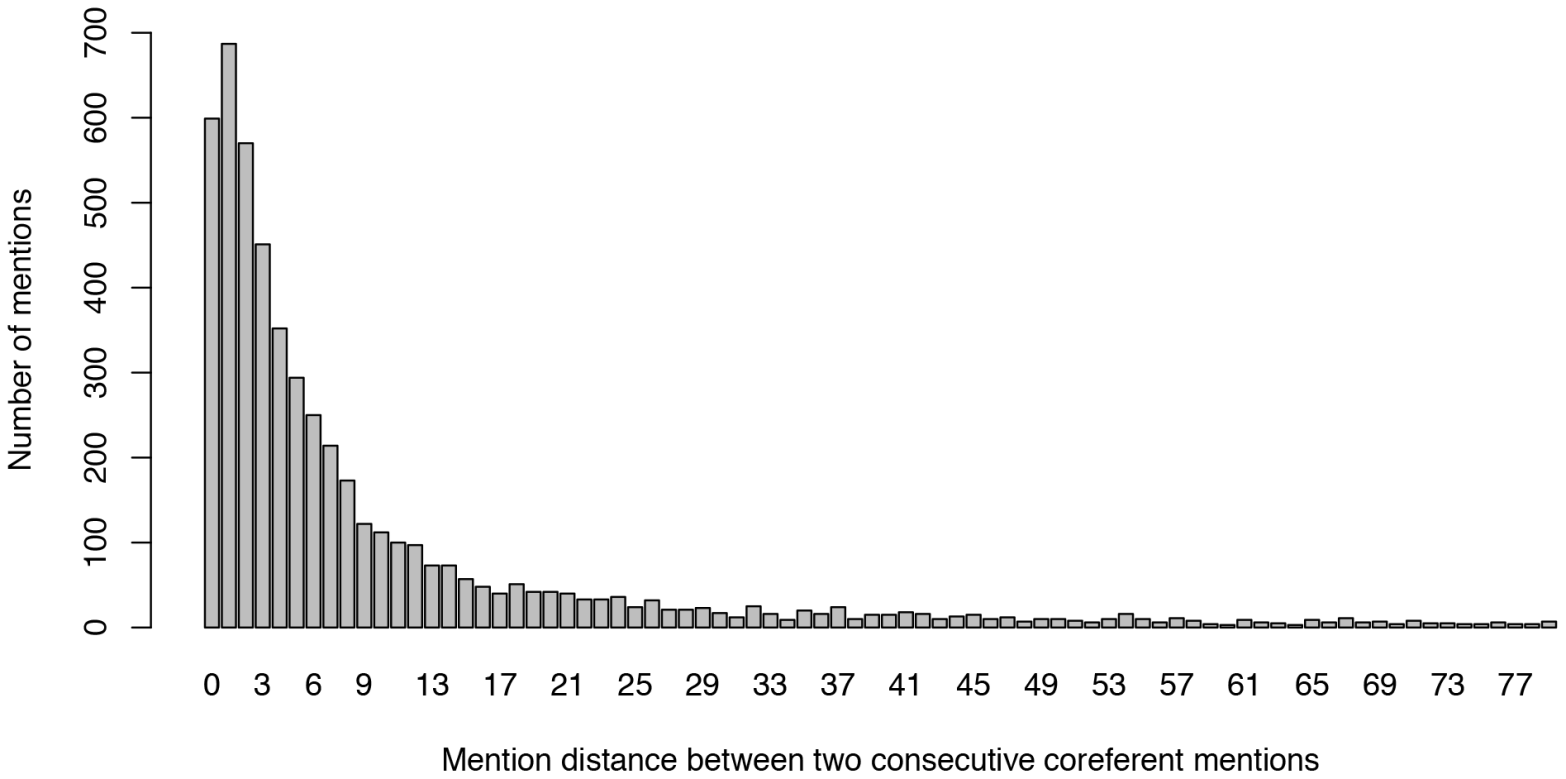
Distribution of distances between two consecutive coreferent mentions. The data was taken from the SemEval2010 [46] coreference dataset. Distance 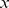 between two consecutive mentions means that there exist 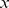 other mentions between them.

**Figure 3:**
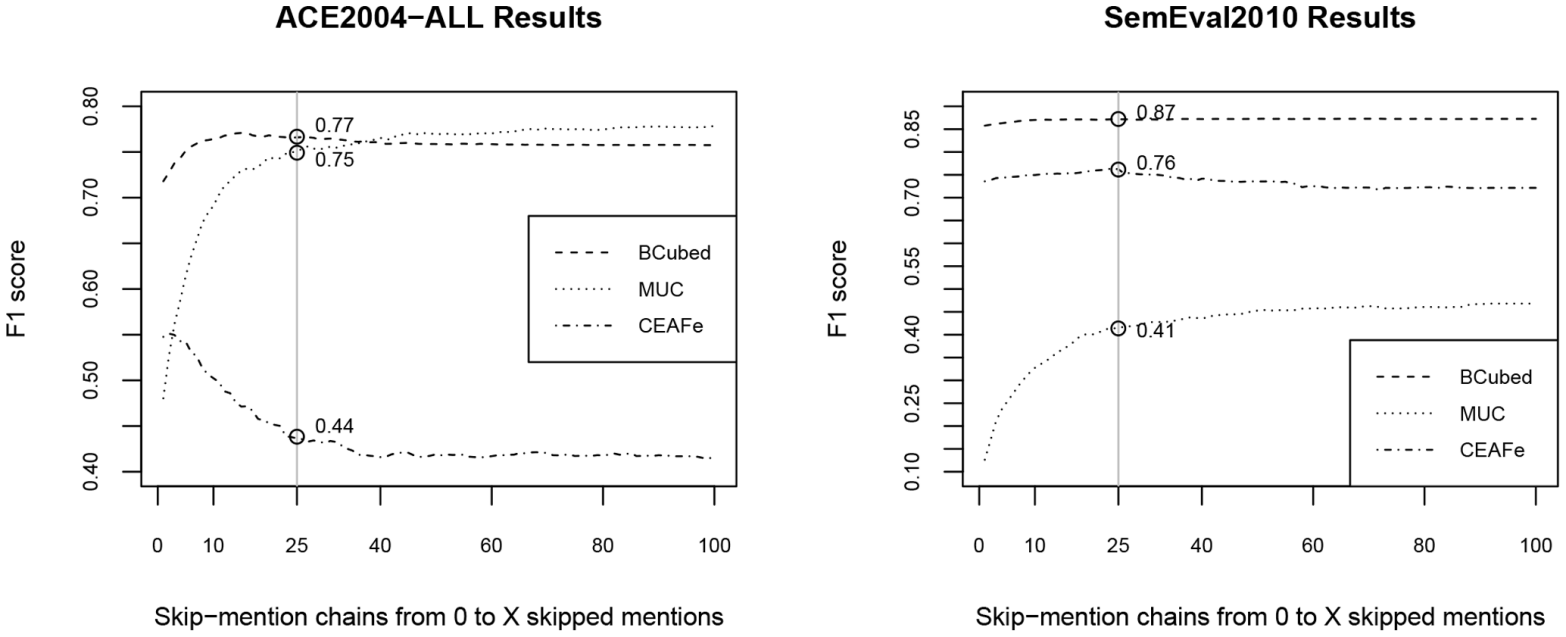
Coreference resolution results using different skip-mention sequences. Evaluation of the proposed system on the whole ACE2004 [45] and SemEval2010 [46] datasets using the metrics BCubed [41], MUC [9] and CEAFe [42].

To support our transformation idea, we show the distribution of distances between two consecutive coreferent mentions (see Figure 2) in the SemEval2010 evaluation dataset. Although the figure shows the distribution for only one dataset, it is representative enough to illustrate the general problem, which is the same for all other datasets. According to the distribution, only 10% of the directly consecutive mention pairs are coreferent, while the highest number (i.e., 12.5%) of coreferent mention pairs are at distance one - i.e., having one other mention in between. Taking into account all mention pairs up to a distance of 20, cumulatively, 81% of the mention pairs can be identified. With distances up to 50, about 92% of the mention pairs can be identified. However, by using longer or all possible distances, the accuracy of a general coreference system is not expected to increase since more false positives are extracted. To overcome such problems, a promising cut-off point is selected (see Figure 3).

Thus, to detect coreferences we form a zero skip-mention sequence from each document, which contains all the mentions from a document. Then we form specific 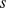 skip-mention sequences. Each 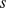 skip-mention sequence contains every 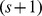-th mention from a document and one linear-chain CRF model is trained for each value of 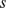. In the next section we present an example of detecting coreferences using skip-mention sequences.

### A Worked Example

In this section we illustrate the detection of coreferences using our approach from the following document: “John is married to Jena. He is a mechanic at OBI and she works there. It is a DIY market.”. Let 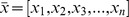 denote a sequence of all mentions within the document. Mentions 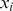 are ordered by their occurrence in the document. For example, from the document we select all entity mentions into one training mention sequence 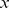:
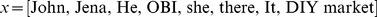
(3)


As mentions mostly consist of noun phrases we could also identify a mechanic as a mention. Due to the simplification of the process the phrase was not identified as a mention during the mention detection. Our goal is now to detect the target clusters for each entity 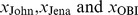:
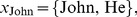
(4)

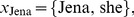
(5)

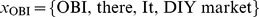
(6)


In some cases, a mention could overlap with another mention. We treat such pairs as separate mentions and order them lexicographically by the index of the first word and mention length.

**Figure 4:**
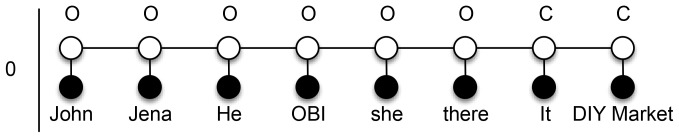
Zero skip-mention training sequence. Initial mention sequence that contains all mentions from the input text “*John* is married to *Jena*. *He* is a mechanic at *OBI* and *she* works *there*. *It* is a *DIY market*.” If the current mention is coreferent with the previous one, it is labeled with *C*, otherwise with *O*.

First, we decide to use zero, one and two for 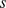 skip-mention sequences and this is also a parameter to the system. In Figure 4, we show a training mention sequence 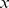, which is applicable to first-order probabilistic models. We call it a ‘zero skip-mention sequence’ because it includes all mentions from a document and there are no (i.e., zero) other mentions between any two consecutive mentions in it. To identify coreferent mentions in the sequences, we need to label them using the labels 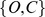. The label 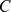 states that the current mention is coreferent with the previous one, whereas 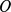 states that the current mention is not coreferent with the previous one. Our linear-chain CRF models are learned over these labels and are therefore able to infer new labels for unseen mention sequences. Observe that for the toy example above, first-order models detect just three coreferent mentions 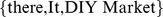 from a zero skip-mention sequence.

To solve the problem of identification of coreferent mentions at longer distances that contain other mention in between (e.g., OBI and there), we introduce further transformations. All additional skip-mention sequences are generated from the initial zero skip-mention sequence 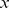 and are labeled accordingly using 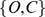 labels. We also train a separate linear-chain CRF model for each additional skip-mention sequence type, which enables us to tag new unseen data for specific skip-mention distance.

**Figure 5:**
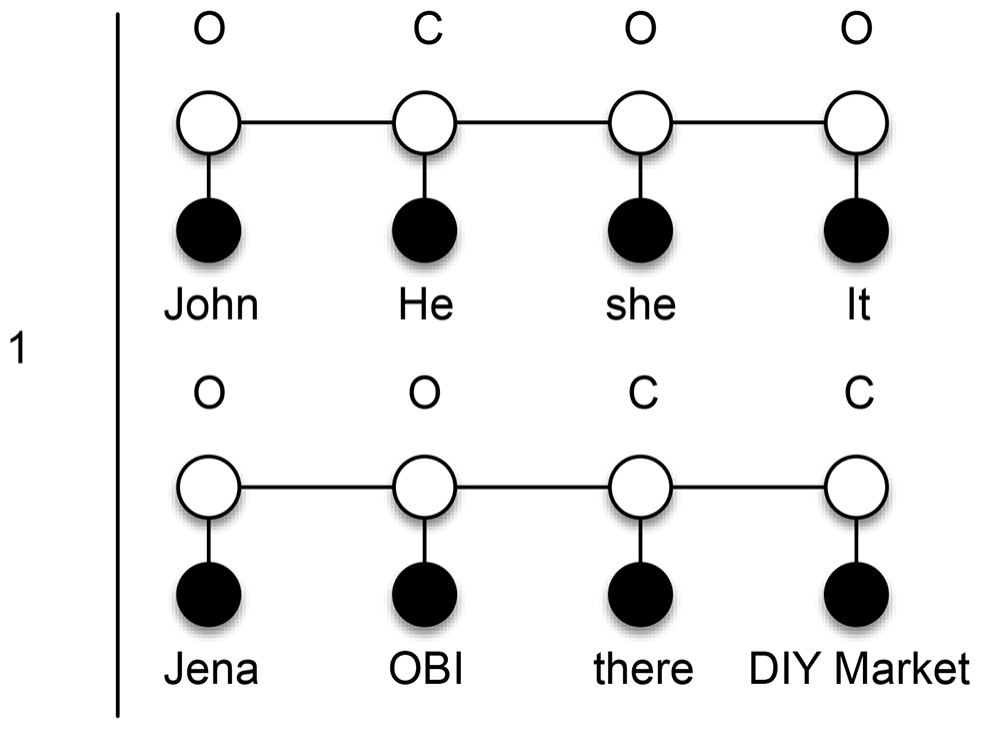
One skip-mention training sequences. Mention sequences that include every second mention (i.e., one skip-mention) from the input text “*John* is married to *Jena*. *He* is a mechanic at *OBI* and *she* works *there*. *It* is a *DIY market*.” If the current mention is coreferent with the previous one, it is labeled with *C*, otherwise with *O*.

**Figure 6:**
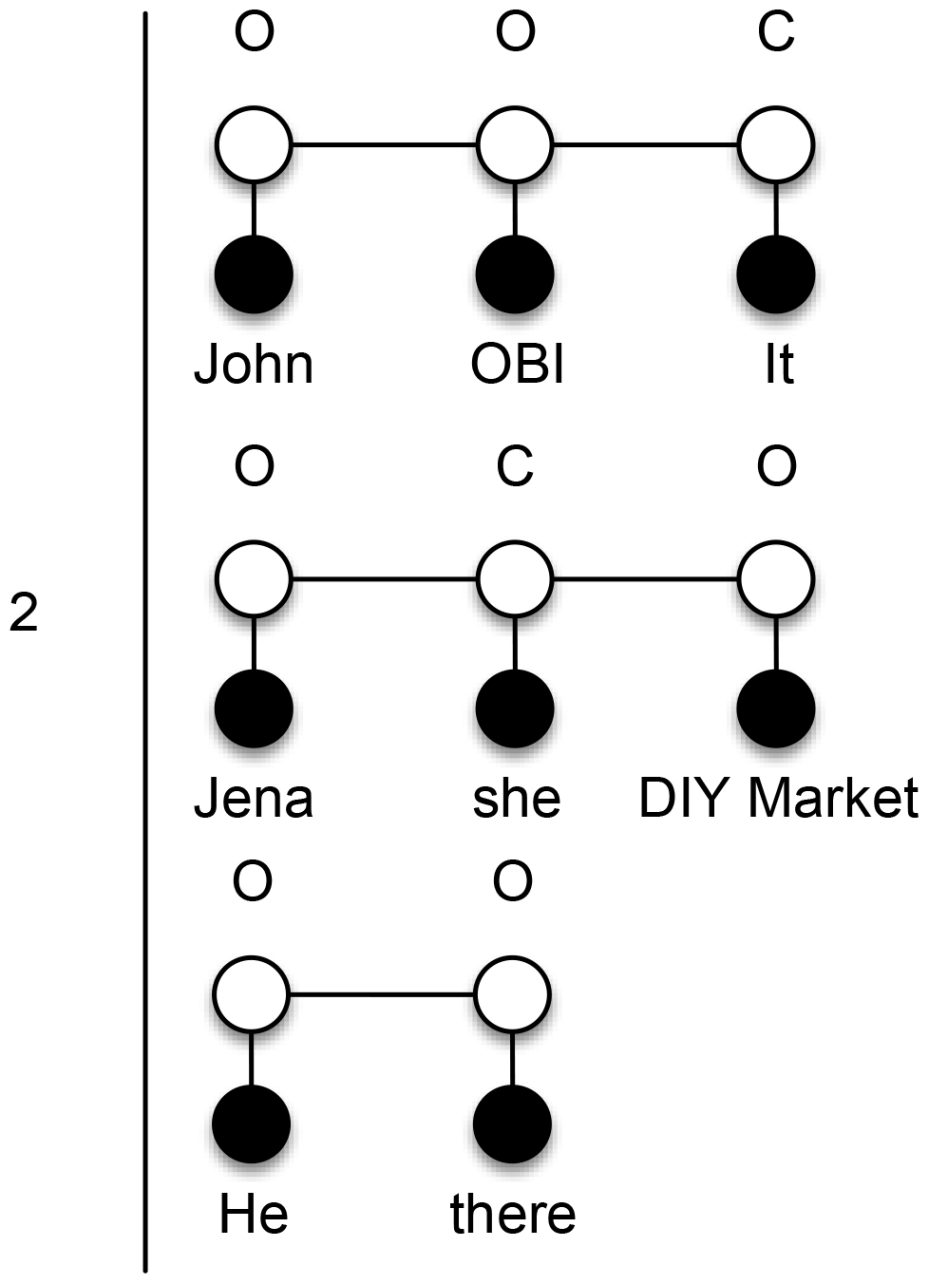
Two skip-mention training sequences. Mention sequences that include every third mention (i.e., two skip-mention) from the input text “*John* is married to *Jena*. *He* is a mechanic at *OBI* and *she* works *there*. *It* is a *DIY market*.” If the current mention is coreferent with the previous one, it is labeled with *C*, otherwise with *O*.

Next, we then generate one skip-mention sequences (see Figure 5), which contain every second mention from the 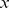 above. The trained model for one skip-mention sequences can therefore extend our results by two new pairs 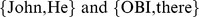. Analogously, for the two skip-mention sequences (see Figure 6) we could get our final missing pairs 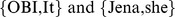.

Lastly, we perform mention clustering from the previously extracted results from all the skip-mention sequences and return target entity clusters 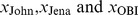.

As shown in the example above, the transformation into higher skip-mention sequences returns more sequences per document. Intuitively, at distance zero, we get one training sequence per document (it contains all document mentions). At distance one, we get two sequences (each contains every second mention). At distance two, we get three sequences, etc. Therefore, the transformation into 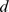 skip-mention sequences returns 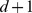 sequences of length 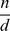, where 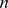 is the number of all mentions in the document.

### The SkipCor System

**Figure 7:**
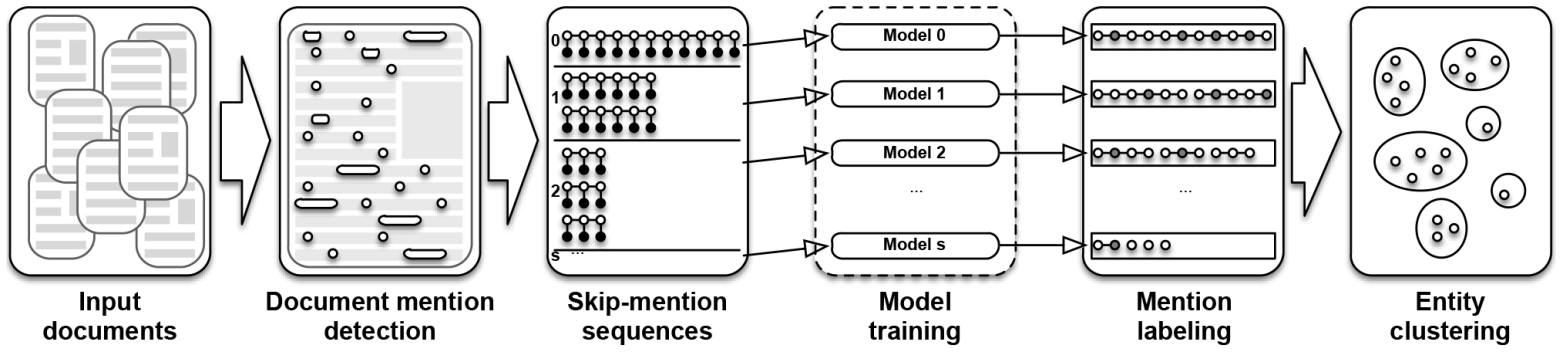
High level skip-mention coreference resolution data flow. The input to the system is given as a set of documents. For each document we select mentions and transform them into mention sequences. According to the system parameters, sequences contain every *s+1*th mention (i.e., *s* skip-mention). A model is trained for each sequence type and then used for labeling. After sequences are labeled, the mentions are then clustered. Each cluster of mentions represents a specific entity, which is also the final result of the system.

The SkipCor system takes a set of documents as input and returns a set of coreferent mention clusters, where each cluster represents an entity to which the mentions refer. The algorithm first reads mentions from the text and then transforms them into skip-mention sequences. Then, we load LCRF models specific to the generated skip-mention sequences and each of these independent models returns separately tagged skip-mention sequences, which are used at the clustering step. The final result is therefore a set of entities (represented as clusters of mentions) for each input document. We show a high level SkipCor data flow in Figure 7 and the detailed algorithms for training and inference are presented in Table 3 and Table 4, respectively.

**Table 3:** Algorithm 1.

Algorithm 1: Skip-mention classifier training
**Input**: training documents D, feature functions 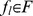 and skip-mention distances 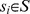
**Output**: skip-mention model 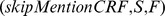
1: 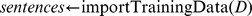
2: 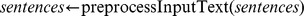
3: 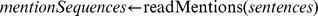
4: 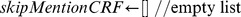
5: **parallel for each** 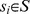 :
6: 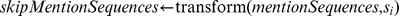
7: 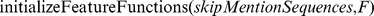
8: 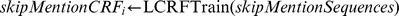
9: **return** 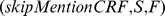

**Table 4:** Algorithm 2.

Algorithm 2: Skip-mention classifier labeling
**Input**: document 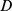 and a skip-mention model ( 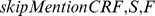 )
**Output**: coreferent mention clusters
1: 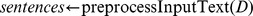
2: 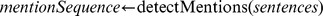
3: 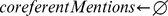
4: **parallel for each** 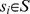 :
5: 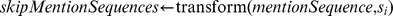
6: 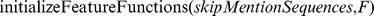
7: 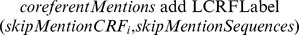
8: 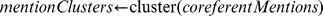
9: **return** 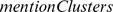

The training phase is similar to the inference phase. The only difference is that the training must occur before any inference (the dashed rectangle in Figure 7). Each of the trained LCRF models is then able to infer the labels for a specific skip-mention distance.

During the training phase, Table 3, we build a skip-mention coreference resolution model. The algorithm takes as input the training documents, a list of feature functions, and a list of skip-mention distances. First, in the pre-processing step, we import the training data in the form of sentences and enrich them with additional tags (e.g., part-of-speech tags, lemmas, parse trees). Then we generate mention sequences (i.e., with zero skip-mentions) for each document. These sequences contain references to the original sentences, therefore the feature functions can use context data from the original input text and not only from the mention sequences. The main part of training the algorithm is the for loop, in which we transform the original mention sequences into the appropriate 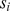 skip-mention sequences, generate features, and train a specific model for every 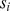 using the LCRFTrain function. Each for loop execution is independent of the others, thus, the algorithm can be parallelized. Lastly, the final result of training is a SkipCor model, which is a tuple consisting of a list of trained skip-mention linear-chain CRF models, a list of the corresponding skip-mention distances, and a list of the feature functions.

To detect coreferences in unseen documents, we follow the algorithm shown in Table 4. As input, we take a raw text document and a SkipCor model that was trained using the algorithm in Table 3. During the execution, similarly to the training phase, we preprocess the input document and generate the initial mention sequence. If the mentions were not already detected in the input document, we perform a rule-based mention detection [14] to generate the initial mention sequence. Due to fact that we are processing only one document, we get only one zero skip-mention sequence at this step (line 2). In the parallel for loop, we transform the initial mention sequence into 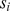 skip-mention sequences, generate the features, and execute the labeling of the specific 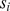 skip-mention LCRF model. All mention pairs that are identified as coreferring are stored in a set, which is the result of the parallel for loop. Lastly, during the clustering step we merge the coreferent mentions into mention clusters, where each cluster represents an underlying entity. These entity clusters are returned as the final result of the SkipCor coreference resolution.

The clustering step is performed using hierarchical agglomerative clustering. All the identified coreferent pairs that were extracted from the labeled zero-skip mention sequence are represented as initial mention clusters. If a mention is coreferent to no other mentions, it will form a singleton cluster. The initial clusters are then iteratively merged according to other labeled 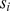 skip-mention sequences. The final result of clustering is also the final result of the SkipCor labeling, and consists of a set of clusters that represent separate entities.

The time complexity of both proposed methods is mainly determined by the training and inference of the LCRF models (i.e., LCRFTrain and LCRFLabel), since other routines can be run in linear time. Still, some third-party methods used at pre-processing could consume more time. Due to the parallel execution of the for loop, we need to find the longest lasting execution. Let us say that the CRF training or inference has a time complexity of 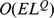 [38], where 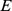 is the number of edges in the graph, 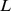 is the number of labels, and 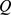 is the size of the maximal clique. In our type of CRF model, we use two possible labels: 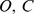, and the size of every clique is two. The number of edges 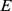 depends on the sequence input to the algorithm. Let us say that there are 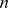 mentions in a document, which results in a zero skip-mention sequence with 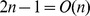 edges. Moreover, every other generated 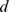 skip-mention sequence contains 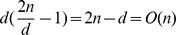 edges. Thus, we conclude that by employing parallelization, CRF models would use 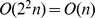 of time. Additionally, next to other linear time procedures, it is also important to include the time for feature function initialization, which takes on the order of 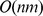, where 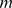 is the number of input feature functions.

## Results and Discussion

In this section, we first explain the coreference resolution evaluation metrics, the system settings that are used during the analysis, and give an overview of the SkipCor baseline systems. Then we introduce the evaluation datasets with some general statistics, labeling specifics, and additional attributes used for training. Next, we show the evaluation results on all the datasets, compare the SkipCor system to two baseline systems, and discuss the results. Lastly, we see how the system accuracy drops when training it on one dataset and testing it on another, to show the expected accuracy in real life scenarios.

### Experimental Framework

There is no general agreement on which metric to use for the coreference resolution task. We here adopt the measures most commonly used in the literature, which will be described below. Prior to the measures we use in this paper, a graph based scoring algorithm had been used, that produced very unintuitive results [39], [40]. There have been a number of metrics proposed, so we evaluate the system using the following most commonly used measures:

#### MUC

The key idea in developing the MUC measure [9] was to give an intuitive explanation of the results for coreference resolution systems. It is a link-based metric (it focuses on pairs of mentions) and is the most widely used. MUC counts false positives by computing the minimum number of links that need to be added in order to connect all the mentions referring to an entity. Recall, on the other hand, measures how many of the links must be removed so that no two mentions referring to different entities are connected in the graph. Thus, the MUC metric gives better scores to systems having more mentions per entity, while it also ignores entities with only one mention (singleton entities).

#### BCubed

The BCubed metric [41] tries to address the shortcomings of MUC by focusing on mentions, and measures the overlap of the predicted and true clusters by computing the values of recall and precision for each mention. If 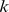 is the key entity and 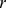 the response entity containing the mention 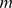, the recall for mention 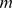 is calculated as 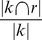, and the precision for the same mention, as 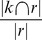. This score has the advantage of measuring the impact of singleton entities, and gives more weight to the splitting or merging of larger entities.

#### CEAF

The goal of the CEAF metric [42] is to achieve better interpretability. The result therefore reflects the percentage of correctly recognized entities. We use entity-based metric (in contrast to a mention-based version) that tries to match the response entity with at most one key entity. For CEAF, the value of recall is 
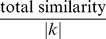
, while precision is 
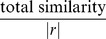
.

For the evaluation in this paper, only exact mention matches are considered as correct, see [43] with some modifications proposed by Cai and Strube [5].

The majority of the state-of-the-art systems were evaluated on specialized shared tasks at MUC (Message Understanding Conference) [44], ACE (Automatic Content Extraction) [45], SemEval2010 (Semantic Evaluation) [46], and, most recently, at CoNLL-2011 and CoNLL-2012 (Conference on Computational Language Learning) [43], [47]. Some general information regarding the English datasets that we used in our evaluation is shown in Table 5. We focused primarily on newswire and broadcast news texts, which have been the most thoroughly studied in the past. To be more specific, we used the following datasets: (1) The ACE 2004 dataset, which in addition to broadcast news and newswire texts, also contains transcripts of conversations and various news reports transcribed and translated from Chinese and Arabic. It is the de facto standard dataset for all major information extraction tasks. (2) The SemEval 2010 dataset was designed specifically to evaluate coreference resolution systems in six languages. The English section of the dataset contains newswire and broadcast news from The Wall Street Journal and the TDT-4 collection. (3) The CoNLL 2012 corpus is one of the largest coreference resolution datasets. It tries to provide a much larger selection of coreferring entities, connecting together events and entities. The corpus consists of newswire texts, magazine articles, broadcast news, broadcast conversations, web data, conversational speech, and an English translation of the New Testament.

**Table 5:** Dataset descriptions.

Dataset	# documents	# sentences	# tokens	# mentions	# entities
ACE2004-ALL	450	7,518	191,387	29,724	12,439
ACE2004-NW	127	2,865	74,987	11,188	4,701
ACE2004-BN	220	3,782	71,602	11,323	4,918
SemEval2010-Train	229	3,648	78,831	21,550	16,082
SemEval2010-Test	85	1,141	24,121	6,692	4,839
CoNLL2012-ALL-Train	1,914	75,185	1,299,310	154,760	33,113
CoNLL2012-ALL-Test	221	9,479	169,579	19,677	4,217
CoNLL2012-NW-Train	734	15,288	387,082	34,470	9,404
CoNLL2012-NW-Test	88	1,898	49,235	4,361	1,168
CoNLL2012-BN-Train	748	9,723	180,300	22,262	6,433
CoNLL2012-BN-Test	93	1,252	23,209	2,936	790

The acronyms ALL (i.e., whole), NW (i.e., newswire), BN (i.e., broadcast news) stand for different subdatasets of the whole dataset, which is further divided into training and test portions.

The proposed system is trained to detect coreferences over all tagged mention types: named, nominal, and pronominal. Due to differences in annotator agreements and rules for tagging the mentions, we cannot compare the results between the corpora. For example, the ACE and CoNLL datasets both include tags for all three mention types, but CoNLL includes more general entities. The CoNLL dataset also includes exact mention phrase boundaries, without considering parse tree constituents (a subtree that identifies an exact token sequence). Therefore it is expected for the results to be lower on CoNLL. Furthermore, SemEval includes only nominal mention types and heuristically identified singleton mentions. Nevertheless, we still conducted additional experiments involving training on one dataset type or domain and testing on another. We will present these results since the main motivation for the whole IE field is to develop techniques that work on an unpredictable user text input, where a user does not know what kind of data the algorithms were trained on.

To get additional annotations for the datasets, we used Apache OpenNLP toolkit [48] sentence splitter, POS tagger, and a dependency parser. For the LCRF training and inference, we used CRFSuite [49] with a cut-off threshold of three features and a default setting, which uses the L-BFGS optimization method. The whole implementation along with the evaluation of the proposed skip-mention coreference resolution is available in a public source code repository [31].

### Empirical Comparisons

As already mentioned, the accuracy of the system depends on the skip-mention sequence types: the accuracy may not increase when using larger and larger skip-mention distances. In Figure 3, we show the results of training the models using different skip-mention sequence distances. From the results, we observe that when taking into account skip-mention distances larger than 40, the F1 scores do not increase or change significantly because although the recall scores increase, the precision scores decrease. Therefore, the final F1 score remains almost stalled due to a compensation of both scores, or even starts to slightly decrease. The scores that we further present in the evaluation were recorded using all skip-mention distances from zero to 25 (cut-off lines in Figure 3). We did not perform any mention detection, and therefore we always compare the results to the settings with already detected mentions.

We compared the proposed SkipCor system to the baseline systems SkipCorZero and SkipCorPair, both using the same feature functions and settings as SkipCor. The only difference between them is the use of different skip-mention sequence types. SkipCorZero detects coreferences only over zero skip-mention sequences, while SkipCorPair checks every mention pair within a document and predicts whether the two mentions are coreferent or not. Due to the large number of mention pairs considered by SkipCorPair, we limited the distance of the mention pairs to ten mentions. SkipCorPair therefore consists of ten LCRF models, each of which is trained to label coreferentiality on skip-mention sequences of length of two mentions.

In Table 6 we present the results for the ACE2004 dataset. When using the newswire and broadcast news portion, we split the data into training and testing sets in the ratio 70∶30. For the whole ACE dataset, we used 336 documents for training and the others for testing [12]. SkipCorZero and SkipCorPair achieved relatively good or best precision values but very low recall. Generally, SkipCorPair outperformed SkipCorZero, while the proposed SkipCor system outperformed both of them. In comparison to other proposed systems, SkipCor achieved a slightly better BCubed score but a lower MUC score. As the results are so close, and opposite for the two measures, it is hard to decide which system is better. On broadcast news, we achieved better MUC and BCubed scores, which are similar to the ones from the newswire section. On the other hand, the precision values are lower, but we achieved a lower difference between the precision and recall compared to the competitive systems. Therefore, we uncovered a lot more mention clusters that have more errors, but the overall results are better. Lastly, we tested the system over the whole dataset (ACE2004-ALL), where we achieved results comparable to those of other systems.

**Table 6:** Results of the proposed SkipCor system, baseline systems, and other approaches on the ACE2004 datasets.

	MUC			BCubed		
	P	R	F	P	R	F
**System**	**ACE2004-NW**					
SkipCor	78.6	68.8	73.4	75.7	**78.6**	**77.1**
SkipCorZero	78.5	22.6	35.1	**96.3**	51.9	67.4
SkipCorPair	78.2	49.0	60.3	85.3	61.7	71.6
Finkel et al. [55]	78.7	58.5	67.1	86.8	65.2	74.5
Soon et al. [16] 1	**85.3**	37.8	52.4	94.1	56.9	70.9
Haghighi et al. [15]	77.0	**75.9**	**76.5**	79.4	74.5	76.9
Stoyanov et al. [21]	-	-	62.1	-	-	75.5
	**ACE2004-BN**					
SkipCor	76.3	**71.3**	**73.7**	76.2	**81.5**	**78.8**
SkipCorZero	79.3	28.3	41.7	**95.9**	57.3	71.8
SkipCorPair	80.9	59.4	68.5	86.3	70.7	77.7
Finkel et al. [55]	87.8	46.8	61.1	93.5	59.9	73.1
Soon et al. [16] 1	90.0	43.2	58.3	95.6	58.4	72.5
	**ACE2004-ALL**					
SkipCor	79.5	70.9	75.0	76.3	**81.1**	78.6
SkipCorZero	**81.3**	28.9	42.6	**95.6**	55.4	70.2
SkipCorPair	80.5	57.1	66.8	84.8	68.9	76.0
Cullota et al. [12]	-	-	-	86.7	73.2	79.3
Bengston et al. [17]	-	-	-	88.3	74.5	**80.8**
Haghighi et al. [15] 2	74.8	**77.7**	**76.2**	79.6	78.5	79.0

Coreference resolution systems evaluated on the ACE2004 dataset (i.e., ALL) [45] and its newswire (i.e., NW) and broadcast news (i.e., BN) subdatasets using the metrics MUC [9] and BCubed [41].

1 Results were reported by Finkel and Manning [42].

2 The MUC F1-score value does not agree with reported precision and recall and has been recalculated.

The results for the CoNLL2012 dataset are shown in Table 7. The corpus is already separated into training, testing, and development datasets (we did not use the last when training). We used gold mention boundaries and additional manual tags, which are included in the data, therefore the results are comparable to the Gold Mention Boundaries setting. Fernandes et al. [34] proposed the shared task winning system and they are also the only ones who published their results on the broadcast news and newswire subdatasets (i.e., CONLL2012-BN, CONLL2012-NW). Similarly to the ACE2004 results, SkipCor performed better than SkipCorPair and SkipCorZero, except on the CoNLL2012-BN subdataset, where SkipCorPair outperformed SkipCor as it achieved the best precision and good recall. Otherwise, on most of the measures, SkipCor slightly outperformed the other systems and achieved better results with the MUC metric, generating cleaner mention clusters. For the full shared task, nine research teams submitted their results, but we show the results of only the top six. We significantly outperformed the others according to the MUC metric, where we increased the precision while having a comparable level of recall. According to the BCubed metric, the results are very similar, but in terms of CEAF we performed a little worse. The systems at the shared task were ranked using the CoNLL2012 measure, which is an average score of the MUC, the BCubed, and the CEAF 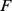-scores. The winning Fernandes et al. [34] system achieved a CoNLL2012 score of 63.1 on English data, whereas our system achieved a score of 61.3, ranking as the second. The next then got the score of 60.7, with the others ranging down to the score of 43.0.

**Table 7:** Results of the proposed SkipCor system, baseline systems, and other approaches on the CoNLL2012 datasets.

	MUC			BCubed			CEAF		
	P	R	F	P	R	F	P	R	F
**System**	**CoNLL2012-NW**								
SkipCor	76.1	**61.5**	**68.0**	69.6	**70.9**	**70.2**	39.9	**59.8**	**47.8**
SkipCorZero	76.7	16.8	27.5	**95.5**	36.7	53.0	35.1	35.3	35.2
SkipCorPair	**81.3**	45.9	58.7	86.4	56.7	68.5	**42.0**	54.7	47.5
Fernandes et al. [34]	-	-	61.6	-	-	70.0	-	-	46.6
	**CoNLL2012-BN**								
SkipCor	76.2	**62.0**	**68.4**	69.8	**71.3**	**70.5**	34.2	**59.2**	43.3
SkipCorZero	76.8	19.4	31.0	**95.9**	45.8	62.0	37.4	37.6	37.5
SkipCorPair	**81.9**	44.7	57.8	88.6	54.6	67.6	**39.1**	56.3	46.1
Fernandes et al. [34]	-	-	65.6	-	-	70.0	-	-	**49.1**
	**CoNLL2012-ALL**								
SkipCor	84.9	63.6	**72.7**	74.6	65.6	69.8	32.9	**57.2**	41.7
SkipCorZero	81.8	21.7	34.3	**95.7**	39.7	56.1	32.1	32.7	32.4
SkipCorPair	**85.9**	50.7	63.8	86.5	53.4	66.0	30.8	53.8	39.2
Fernandes et al. [34]	77.5	64.9	70.7	79.0	64.3	**70.9**	**41.7**	56.5	**48.0**
Björkelund et al. [56]	71.6	63.4	67.3	76.6	64.0	69.7	41.4	50.0	45.3
Chen et al. [57]	66.8	63.3	65.0	73.6	65.4	69.2	44.9	48.8	46.8
Stamborg et al. [58]	58.8	**66.2**	62.3	65.0	**71.2**	68.0	45.8	38.5	41.8
Zhekova et al. [51]	54.7	55.0	54.8	55.6	61.9	58.6	34.7	34.4	34.5
Li et al. [59]	33.7	44.2	38.2	53.9	66.4	59.5	36.5	27.5	31.4

Coreference resolution systems evaluated on the CoNLL2012 dataset (i.e., ALL) [43], and its newswire (i.e., NW) and broadcast news (i.e., BN) subdatasets using the metrics MUC [9], BCubed [41] and CEAF [42].

In Table 8 we show the results for the SemEval2010 dataset, which is already separated into training and testing portions. We compared the systems using the Gold-standard Closed setting, for which systems can use only the provided attributes with true mention boundaries. On this dataset, SkipCor outperformed SkipCorZero on all three measures and outperformed SkipCorPair in terms of the CEAF and BCubed metrics. Interestingly, SkipCorPair achieved a significantly higher MUC precision score, and it therefore outperformed SkipCor in this measure. Compared to other systems, SkipCor achieved better BCubed and CEAF scores, but a lower MUC score. Interestingly, in the selected setting, the RelaxCor system performed the best, but our system outperformed it on all three measures. Focusing only on the MUC measure, we got the second place, as the SUCRE system achieved a better recall score.

**Table 8:** Results of the proposed SkipCor system, baseline systems, and other approaches on the SemEval2010 dataset.

	MUC			BCubed			CEAF		
	P	R	F	P	R	F	P	R	F
**System**	**SemEval2010**								
SkipCor	68.8	30.1	41.8	94.8	**80.8**	**87.3**	74.0	78.5	**76.2**
SkipCorZero	67.0	3.6	6.8	**99.6**	75.1	85.7	73.0	73.1	73.1
SkipCorPair	**76.7**	35.6	48.7	97.1	79.0	87.1	72.7	**79.4**	75.9
RelaxCor [60]	72.4	21.9	33.7	97.0	74.8	84.5	**75.6**	75.6	75.6
SUCRE [61]	54.9	**68.1**	**60.8**	78.5	86.7	82.4	74.3	74.3	74.3
TANL-1 [33]	24.4	23.7	24.0	72.1	74.6	73.4	61.4	75.0	67.6
UBIU [50]	25.5	17.2	20.5	83.5	67.8	74.8	68.2	63.4	65.7

Coreference resolution systems evaluated on the SemEval2010 dataset [46] using the metrics MUC [9], BCubed [41] and CEAF [42].

System UBIU [50], which entered the SemEval2010 shared task, also competed at the CoNLL2012 task, with a few modifications [51]. Our system significantly outperformed UBIU on both tasks and in terms of all three metrics. In contrast to our proposal, UBIU uses pairwise classification with a form of memory-based machine learning.

According to the results we showed, SkipCor outperformed both SkipCorZero and SkipCorPair. SkipCorZero mostly achieved good precision but very low recall. This is due to the identification of coreferences only between consecutive mentions within a document. SkipCor therefore uses skip-mention sequences to boost the recall values and consequently also the final result. SkipCorPair ranks somewhere between SkipCorZero and SkipCor. It checks for coreferences between mention pairs and is therefore very similar to other pairwise approaches. Due to a lot of pairwise comparisons, many mention sequences of length two must be generated, and therefore SkipCorPair executes more slowly than SkipCor.

Generally, SkipCor showed improvements on most of the datasets or achieved comparable results. We did not asses statistical significance of the differences in accuracy between the various systems because their implementations are not accessible and also referenced papers report single F score values only. Although some of the the existing rule-based systems are easy to implement and achieved good or best results, they may not be easily adapted to a different domain. This is also the reason why we proposed a simple machine-learned method for the task. SkipCor mostly obtained very good recall scores and a little bit lower precision. Other top performance systems use hybrid approaches, combining rule-based strategies with machine learning. All of them also employ feature engineering with a heavy use of lexicalized features. At the ACE2004 task, Haghighi et al. [15] used a completely deterministic approach, driven entirely by syntactic and semantic constraints. Bengston and Roth [17] focused especially on rich feature functions engineering with a simple pairwise classifier based on averaged perceptron. At the SemEval2010 shared task, the best two systems used a combination of manual rules and a set of machine learning classifiers (i.e., decision trees, naive Bayes, SVM, or maximum entropy models). Lastly, the CoNLL2012 task winner, Fernandes et al. [34], looked for the best mention clustering within a document using a specialized version of structure perceptron and represented mention clusters as coreference trees. The only system that used first-order probabilistic models was the one by Cullota et al. [12] on the ACE dataset. Their usage is completely different from that of our approach, because they still perform standard pairwise comparisons and then use first-order logic over mention clusters. Other CRF-based approaches, which were mentioned within the section on related work, were tested only against a limited version of a coreference resolution dataset or focused on an entity resolution task, which is a little similar to coreference resolution.

### Performance in Real-world Scenarios

In addition to standard evaluation techniques, we trained SkipCor on one dataset and tested it on another (Table 9). Although the datasets do not have the same annotation guidelines or domain, this is interesting, as showing the results that can be expected by an end user on real data.

**Table 9:** Comparison of the results when training on one type of dataset or domain and testing on another.

	Model				
Dataset	A-BN	A-NW	C-BN	C-NW	SemEval2010
** A-BN**	*74, 78, 54*	**72**, 77, 39	65, 70, 28	64, 69, 29	42, **71**, 49
** A-NW**	**72**, 73, 42	*73, 75, 58*	60, 64, 27	59, 69, 29	42, 67, **50**
** C-BN**	33, 56, 37	40, 58, 39	*68, 70, 43*	**65**, 70, 27	**57**, 64, 31
** C-NW**	39, 57, 39	41, 59, 41	**67**, 66, 28	*68, 70, 48*	56, 64, 32
**SemEval2010**	19, **82, 70**	23, **85, 74**	39, 76, 40	39, **77, 33**	*42, 87, 76*

Coreference resolution results comparison on ACE2004 (i.e., A), CoNLL2012 (i.e., C) and SemEval2010 newswire (i.e., NW) and broadcast news (i.e., BN) datasets. Each column represents a model trained on a specific dataset, while each row represents a dataset. Values represent 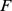-scores of MUC [9], BCubed [41] and CEAF [42], respectively.

First, we notice only a minor performance drop when testing within the datasets from the same shared task. For example, the results between broadcast news and newswire data remained almost the same as for the CoNLL and ACE2004 data separately. Furthermore, CoNLL models performed only a little worse on the ACE2004 dataset than originally. On the other hand, ACE2004 models performed less well on the CoNLL dataset, with a drop of roughly 20%. Both the CoNLL and ACE2004 models achieved low MUC scores on SemEval, but the best BCubed and CEAF scores. The difference is due to the fact that SemEval contains only nominal mentions and heuristically tagged singletons, which are more easily discovered, and they boost the scores. A model trained on SemEval performed the worst on both CoNLL and ACE2004. Interestingly, it achieved better MUC scores on CoNLL data than on the native SemEval testing dataset.

To conclude, the results typically show drops in accuracy on other domains or other datasets of the same or a different domain, from their performance on the same dataset. A similar analysis on different coreference datasets has also been conducted before [52], and their findings also show that evaluation on the same dataset the models were trained on gives the best results.

## Conclusions

The present paper proposed ‘SkipCor’, a novel skip-mention coreference resolution system that is based solely on the linear-chain conditional random fields algorithm. To support the identification of all coreferent mentions in the text, the basic algorithm was extended with an adequate transformation of the data into different skip-mention sequences. In contrast to traditional approaches, the proposed system avoids checking all possible pairwise comparisons or using a single model. Thus, the system is completely parallelizable with a linear time complexity. Due to the amount of textual data available to date, the latter is of considerable importance in practical applications. We also stressed that the proposed skip-mention sequences could be adopted within other approaches in a straightforward fashion, which represents a prominent direction for future research.

The proposed system was evaluated on standard coreference resolution datasets that are the focus of evaluations for the majority of the techniques in the field. We compared the system to some baseline algorithms and also to the best performing coreference systems reported in the literature. The results obtained are comparable to the current state-of-the-art in coreference resolution, while we also more thoroughly analysed the contribution of the proposed skip-mention sequences. In addition, the analysis revealed that although accuracy in real-world scenarios can be even larger than expected, it decreases significantly when the system is trained on less reliable datasets.

Future work will focus on the development of more intelligent SkipCor mention clustering techniques (e.g., weighted scoring of coreference models) to minimize the number of merged conflicting mentions. Moreover, the system will be extended with a domain ontology that will provide an additional source of feature functions.
